# Supplementary material for: Preparation and Properties of Acetoacetic Ester-Terminated Polyether Pre-Synthesis Modified Phenolic Foam
Source: Materials (Basel). 2019 Jan 22;12(3):334. doi: 10.3390/ma12030334 (PMC6384588; doi:10.3390/ma12030334)
Supplement: Supplementary file 1 [file materials-12-00334-s001.pdf]

Table S1. The effect of the acetoacetic ester-terminated polyether with different contents on the properties of the foam.

	**Basic foam**	**Modified foam**					
		10%	12%	14%	16%	18%	20%
Bending strength (MPa)	0.220	0.254	0.276	0.291	0.305	0.296	0.291
Compressive Strength (MPa)	0.118	0.133	0.16	0.184	0.203	0.194	0.185
Fracture Displacement (mm)	4.31	7.1	10.9	14.3	14.8	13.9	11.8
Pulverization Rate (%)	5.7	3.3	2.1	1.6	1.3	1.9	2.1
Apparent Density (g/cm^3)	0.050	0.034	0.036	0.041	0.043	0.049	0.053
Water Absorption Rate (%)	8.0	6.4	6.4	6.3	6.5	6.9	7.3
